# Lancing the Gums of Infants

**Published:** 1865-09

**Authors:** Henry S. Chase


### LANCING THE GUMS OF INFANTS.

BY HENRY S. CHASE, M. D., D. D. S.

Read before the State Dental Society.

Lancing the gums of infants to procure a more speedy eruption of the milk teeth was formerly very common among doctors of medicine. Perhaps it is now, but I think it not so frequent as formerly. Dentists are often called upon to perform this operation, and in many cases where it is not only unnecessary, but absolutely productive of harm.

It should never be done unless we are quite sure that the teeth are *ready* to be erupted. It should not be done unless the gums are red, puffy and swollen.

It should not be done merely because the child is fretful, nervous, troublesome, and the *mother wishes* the operation performed. And here let me say that no dentist should ever disgrace his profession by performing an operation which his sound judgment disapproves, merely to gratify the wishes of the patient, or another, or for the sake of a fee.

In lancing the gums of a child sit down in a common chair: let another person sit close to and facing you. Let the child sit on the knees of the other person, face to face. Bend the child backwards holding its head between your knees, its hands being held by the opposite person. With your left hand you can open the mouth of your little patient and use the lancet with the other. You can now have perfect control of the child and its struggles will be in vain. Let the lancet be *very* sharp, and cut gently, but firmly, down to the teeth, if within reasonable distance; if they are not, don't attempt to reach them. For although to release the struggling teeth, it is necessary to touch them with the lance; yet you may have been mistaken in your diagnosis, and the teeth be too deeply imbedded in the jaw to receive aid; and, as the operation is premature, you may do great injury to the immature enamel of the temporary teeth, and great mischief to the sacks of the permanent ones.

I consider it honorable to acknowledge that I once made this mistake, myself, in the commencement of my practice, but never have a second time. And I acknowledge it for your benefit, that you may profit by my experience, and be on your guard.

My little patient did not erupt its lower inscisors, for three months after this operation, and when they come through, they had the precise appearance of having had a slice of enamel cut off of the labial surface for one-fourth of their length. I *felt* the teeth plainly when I performed the operation, and it flashed across my mind at the time that I was fairly, or rather *un*fairly, cutting into the enamel of these teeth. My fingers received the sensation of cutting through soft, wet sandstone.

Since my own mal-operation I have seen several cases of imperfect enamel that I attributed to the same cause, and on enquiring found that the child's gums had been lanced in infancy.

Now, I think, this lancing of childrens' gums for the relief of teething symptoms, in nine cases out of ten, unnecessary, when demanded by the mother, and I almost invariably "put them off," when called upon for this purpose.

